# Comparison of the Antidepressant-Like Effects of Estradiol and That of Selective Serotonin Reuptake Inhibitors in Middle-Aged Ovariectomized Rats

**DOI:** 10.3389/fnagi.2016.00311

**Published:** 2016-12-21

**Authors:** Saloua Benmansour, Luis D. Arroyo, Alan Frazer

**Affiliations:** ^1^Department of Pharmacology, University of Texas Health Science Center at San Antonio, San AntonioTX, USA; ^2^South Texas Veterans Health Care System, San AntonioTX, USA

**Keywords:** middle age, OVX rats, estradiol, SSRI, FST, SERT function, critical period

## Abstract

This study investigated the effect of age and that of the post-ovariectomy (OVX) time interval on the antidepressant (AD)-like effects of estradiol (E_2_) and selective serotonin reuptake inhibitors (SSRIs) in middle-aged (10 month) OVX rats (10m-OVX). Acute or chronic effects of these treatments in 10m-OVX were compared with those (1) in young adult (4-month) OVX rats (4m-OVX) or with older (14-month) OVX rats (14m-OVX), at a short time: 2 weeks post-OVX (+2w) and (2) in 10m-OVX rats after a longer times: 4 or 8 months post-OVX (+4m or +8m). Using *in vivo* chronoamperometry in the CA3 region of the hippocampus, E_2_ at 20 pmol, a dose shown previously to inhibit the serotonin transporter (SERT) in 4m-OVX, had no effect in 10m-OVX+2w. A higher dose of E_2_ (40 pmol) increased T80 value, a measure of serotonin or 5-hydroxytryptamine (5-HT) clearance, and also blocked the ability of fluvoxamine to increase T80. By contrast, estradiol had no effects on SERT function in 10m-OVX+4m, even at a higher dose than 40 pmol. Fluvoxamine slowed 5-HT clearance in 10m-OVX at +2w, +4m and +8m post-OVX as it did in the 4m-OVX. Using the forced swim test, 2 weeks treatment with E_2_ (5 μg/day), a dose shown previously to induce AD-like effects in 4m-OVX, had no effect in 10m-OVX+2w. However, a higher dose (10 μg/day) of E_2_ induced an AD-like effect as demonstrated by significantly increased swimming behavior and decreased immobility. This effect was not seen in 10m-OVX+4m. By contrast, significant AD-like effects were obtained in 14m-OVX+2w, thereby demonstrating that the lack of an AD effect of E_2_ is due to the 4-month hormone withdrawal and not to an age effect. After 2 weeks treatment with the SSRI sertraline, similar AD-like effects were obtained in 10m-OVX tested at +2w, +4m or +8m post-OVX as those found in 4m-OVX. Thus, the potency of estradiol to produce effects consistent with inhibition of the SERT was not only decreased in older rats but its effects were markedly diminished the longer hormonal depletion occurred. By contrast, the ability of SSRIs to inhibit the SERT was not affected either by age or the length of hormonal depletion.

## Introduction

The efficacy of antidepressants (ADs) in the elderly suffering from major depressive disorder (MDD) is controversial (Kasper et al., 2005; Nelson et al., 2008). Although tricyclic ADs and selective serotonin reuptake inhibitors (SSRIs) are reported to be effective in clinical trials with older patients (Gareri et al., 2000; Salzman et al., 2002; Blazer, 2003) some authors have claimed that their effectiveness is reduced compared to that found in young adults (Lenze et al., 2008; Sheffrin et al., 2009; Tedeschini et al., 2011). Moreover, it has been suggested that such decline begins at middle age as a modest reduction that becomes more pronounced at senescence (Tedeschini et al., 2011).

MDD occurs about twice as frequently in women as in men (Kupfer et al., 2012), with increased occurrence in middle age, around the menopausal transition (Maartens et al., 2002). In fact, the onset or exacerbation of depressive symptoms has been associated with the time around perimenopause (Maki et al., 2010; Soares, 2013). This has provided a rationale to try either estrogen replacement therapy (ERT) or hormone replacement therapy (HRT) in such patients. Such treatment has been reported to have AD effects in peri- and postmenopausal women (Frey et al., 2008; Soares and Frey, 2010), although this is not a universal finding (Morrison et al., 2004). ADs are also used to treat depression in menopausal women. Some reports indicate that the AD response could be modified not only by aging but also the endocrine condition. Although some find pre-menopausal women are more responsive than post-menopausal women to ADs (Soares et al., 2001; Huang et al., 2008; Pae et al., 2009), this is not a universal finding (Kornstein et al., 2013, 2015). A combination of estrogen and AD is also often used to treat depression in menopausal women. However, there appears to be little evidence that taking SSRIs in conjunction with ERT produces greater improvement in depressive symptoms than that seen with an SSRI alone (Frey et al., 2008).

A possible reason for such discrepancy in results is the time when AD treatment was started in relation to menopause onset. A “window of opportunity” or “the critical period” hypothesis has been postulated for the effects of estrogen (Rocca et al., 2011), which proposes that ERT is only beneficial if administration is begun during a time window in close proximity to menopause (Resnick and Henderson, 2002). Many studies have examined the critical period hypothesis for estradiol’s effects with respect to cognition but only a few clinical studies have investigated the effect of the time of initiation of ERT and/or AD therapy after menopause in mood disorders (Craig, 2013). It has been suggested that menopausal status and old age are predictors of a poor response to AD treatment (Pae et al., 2009).

The aims of the present study were to explore whether the age (4-month, 10-month, and 14-month OVX rats) and/or post-OVX interval (2 weeks versus 4 months or 8 months) influences the AD effect of estradiol and that of SSRIs in middle-aged 10-month OVX rats. Many studies primarily examined the acute effects of estradiol or ADs in middle-aged rats; however, such treatments are given chronically to post-menopausal depressed patients. Therefore, this study examined not only the acute effects of E_2_ and of SSRI, using chronoamperometry to provide a direct measure of the effect of local application of an SSRI into the CA3 region of the hippocampus on serotonin transporter (SERT) function, but also the behavioral consequences of chronic treatments with estradiol and with SSRIs in the forced swim test (FST). The FST provides a behaviorally relevant but more indirect measure of the effect of the treatments on SERT function (Cryan et al., 2002). Chronoamperometry experiments, as stated above, are carried out in the CA3 region of hippocampus. This specific area of hippocampus was selected based on earlier work showing that under our experimental conditions active clearance of exogenous 5-hydroxytryptamine (5-HT) from it was due exclusively to the SERT (Daws et al., 1998). The hippocampus, in general, is an appropriate area to study AD effects on SERT function and the influence of ovarian steroids on such effects. It receives a dense serotonergic innervation, with perhaps some preferential innervation from the median raphe (Jacobs and Azmitia, 1992). There is an abundance of data indicating that the SERT is the key, initial cellular target for SSRIs (Lenox and Frazer, 2002). Also, estrogen is known to affect the anatomy and physiology of the rodent hippocampus (McEwen and Alves, 1999). There are substantial estrogen receptor (ER)α and ERβ protein in the hippocampus in general and in CA3 in particular (Shughrue and Merchenthaler, 2000). In addition, both the anatomy and function of the hippocampus are affected by AD treatment (Castrén and Rantamäki, 2010; Duman and Li, 2012).

## Materials and Methods

### Animals

Ovariectomized (OVX) rats (Sprague-Dawley; 4-month, 10-month, and 14-month-old, Envigo, Indianapolis, IN, USA) were housed on a 12 h:12 h light/dark cycle with lights on at 07:00 and with food and water provided *ad libitum*. All animal procedures were in accordance with the National Institutes of Health Guide for the Care and Use of Laboratory Animals and were approved by the local Institutional Animal Care and Use Committee. All efforts were made to minimize the number of animals used, or stress and discomfort to the animals during the experimental procedure. In general, OVX rats (4-month, 10-month, and 14-month) were used 2 weeks after ovariectomy (to allow recovery after surgery before the start of the experiment). However, in studies investigating the effect of time post-OVX, 10–month-old OVX rats were also used 4 months or 8 months post-ovariectomy.

### Choice of Animal Model

The incidence of regular estrous cyclicity decreases progressively during aging. Between 8 and 12 months of age, female Sprague-Dawley rats display prolonged irregular cycles (Matt et al., 1986), a period named periestropause or recurrent pseudopregnancy and is characterized by high levels of follicle-stimulating hormone (FSH) and luteinizing hormone (LH) in comparison with those in young female rats in diestrus (Bestetti et al., 1991). This stage is analogous to perimenopause or menopause transition in women where menstrual cycle irregularities are related to increased levels in FSH and LH (Prior, 1998). The state of complete loss of reproductive capacity in rodents, called estropause, is characterized by persistent diestrus. Lower levels of estradiol (Bestetti et al., 1991) as well as higher levels (Wise and Ratner, 1980; Gore et al., 2000) were reported in middle-aged rats in persistent diestrus as compared with young females in the same estrous-cycle phase. These differences seem to depend upon the strain of rat, age and sensitivity of the hormone assay. Rat estropause differs from monkey and human menopause in that aged rats retain a much larger number of primary oocytes, resulting in higher estrogen levels at the onset of persistent diestrus than those seen in primates after menopause (Chakraborty and Gore, 2004). To limit this difference, in this study rats entering estropause (10–month-old) were ovariectomized thus providing a model that mimics better both the age and ovarian hormone status of peri- and postmenopausal women.

### Choice of SSRIs Used in Acute and Chronic Studies

For the acute studies, the SSRI fluvoxamine was used in the *in vivo* chronoamperometry experiments as we found that it did not interfere with the electrochemical signal of 5-HT as other SSRIs such as fluoxetine or sertraline did. However, fluvoxamine-induced effects observed in such experiments were not specific to it, but also occurred with another SSRI, citalopram, which was also devoid of electrochemical effects on its own (Benmansour et al., 2009).

For chronic studies of AD-like effects, fluvoxamine was not used because it is very expensive such that the cost for chronic administration would be prohibitive; further, is not the Food and Drug Administration (FDA)-approved in the USA for treatment of depression whereas sertraline is. We have selected a dose of sertraline that has been shown not only to downregulate the SERT but also to produce AD-like effects in the FST when given chronically to male rats (Benmansour et al., 1999; Bilge et al., 2008; Furmaga et al., 2011) as well as to female OVX rats (Benmansour et al., 2016).

### Experimental Design and Drug Treatment

In the acute and chronic estradiol experiments, two middle-aged groups—10 months and 14 months—were used 2 weeks post-OVX. The impact of the length of hormonal depletion was studied in 10-month OVX rats by measuring effects at 2 weeks and 4 months post-OVX.

In the acute and chronic SSRI experiments, two age groups were used—young adult (4-month) and middle-aged (10 month), with both used 2 weeks post-OVX. The impact of the length of hormonal depletion was studied also in 10-month OVX rats by measuring effects at 2 weeks, 4 months, or 8 months post-OVX.

Therefore, there were the following groups in the acute or chronic experiments:

(1) 4-month OVX, used 2 weeks post-ovariectomy (4m-OVX+2w)
(1) 10-month OVX, used 2 weeks post-ovariectomy (10m-OVX+2w)
(1) 14-month OVX, used 2 weeks post-ovariectomy (14m-OVX+2w)
(1) 10-month OVX, used 4 months post-ovariectomy (10m-OVX+4m)
(1) 10-month OVX, used 8 months post-ovariectomy (10m-OVX+8m)

For the chronic estradiol experiments, four cohorts of 13–16 rats each were used, 6–8 controls, and 7–8 estradiol-treated animals (groups 2, 3, and 4). Estradiol (5 or 10 μg/day for group 2) and (10 μg/day for groups 3 and 4) was administered for 2 weeks subcutaneously via implantation of osmotic minipumps. Control groups received vehicle which consisted of 25% EtOH/H_2_O.

For the chronic sertraline experiments, four cohorts of 16 rats each were used, 8 controls and 8 sertraline-treated animals (groups 1, 2, 4, and 5). Sertraline was administered (10 mg/kg/day) subcutaneously via implantation of osmotic minipumps. Control groups received vehicle which consisted of 25% EtOH/H_2_O.

### *In vivo* Chronoamperometry

This was carried out as described previously (Benmansour et al., 2012).

#### Animal Preparation

All groups of Sprague-Dawley OVX rats were anesthetized with chloralose (70 mg/kg)/urethane (700 mg/kg) administered intraperitoneally, after tracheal intubation and placed into a stereotaxic apparatus (David Kopf Instruments). The body temperature of the rat was maintained between 37 and 38°C using a water-circulating heating pad. The scalp was incised and reflected and a hole drilled in the skull at the desired coordinates. A small burr hole was drilled over the posterior cortex for placement of Ag/AgCl reference electrode.

#### Electrode Preparation

Carbon fiber electrodes (30 μm tip diameter, 95–175 μm in length) were coated with Nafion to improve their selectivity, then tested for sensitivity to 5-hydroxyindoleacetic acid (250 μM) and calibrated *in vitro* with 5-HT.

#### Micropipette Preparation

The carbon fiber electrode was positioned adjacent to a four barrel micropipette with the tip separation between 250 and 350 μm. The electrode and the multibarrel micropipette were then attached using sticky wax. Micropipette barrels were filled with 5-HT (200 μM, Sigma-Aldrich, St Louis, MO, USA), fluvoxamine (400 μM, Sigma-Aldrich, St Louis, MO, USA; fluvoxamine was always used at 4× the amount of 5-HT applied), or 17-β-estradiol (E_2_, Sigma-Aldrich, St Louis, MO, USA). Drugs were prepared in 0.1 M phosphate-buffered saline (PBS) and supplemented with 100 μM ascorbic acid. The pH of all solutions was 7.4. All drugs were delivered by pressure ejection in a volume of 20–100 nl, using a PLI-100 reproducible pico-injector.

#### Electrochemical Recordings

The electrode-pipette assembly was lowered into the CA_3_ region of the hippocampus [stereotaxic coordinates (mm) anterior–posterior, -4.10 from bregma; medio-lateral, +3.30 from midline; dorsal–ventral, -3.60 from dura; Paxinos and Watson, 1986]. Chronoamperometric recordings were started 20–30 min after the lowering of the assembly to allow the baseline electrochemical signal to stabilize. High-speed chronoamperometric recordings were made using the Fast-16 system (Quanteon). Oxidation potentials consist of 100 ms pulses of +0.55 V versus Ag/AgCl were delivered one per second; the electrode was held at the resting potential of 0.0 V between measurements. Oxidation and reduction currents were digitally integrated during the last 80 ms of each 100 ms voltage pulse.

**FIGURE 1:**
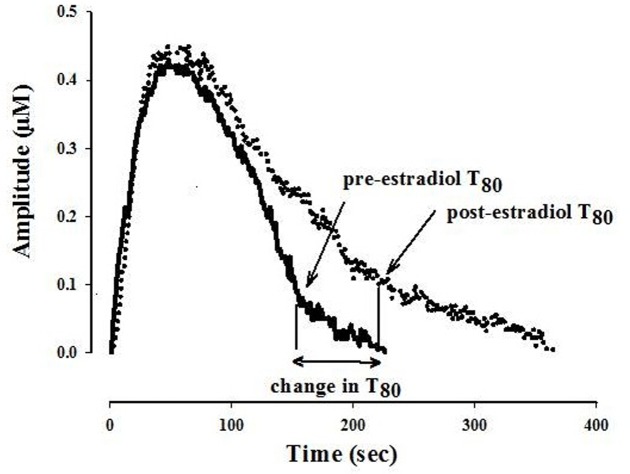
**Representative 5-hydroxytryptamine (5-HT) electrochemical signals illustrating the effect of locally applied 17-β estradiol into the CA3 region of the hippocampus of an OVX rat.** The signal was generated by local application of 5-HT (5.2 pmol). Estradiol 40 pmol was pressure ejected 10 min before the next application of 5-HT. Shown here is the clearance time, T80 parameter: the time it takes for the peak signal amplitude to be reduced by 80%. For clarity, only oxidation signals are shown.

The ability of fluvoxamine to block 5-HT clearance was measured after the 5-HT signal became stable. The effects of estradiol on 5-HT clearance and on fluvoxamine-induced blockade of 5-HT clearance were measured 1–10 min and 40–60 min post-E_2_ application. Several parameters are obtained from the electrochemical signal produced by exogenous applications of 5-HT. Analyzed in this study is the clearance time parameter, T80 which is the time required for the peak amplitude to be reduced by 80% (see **Figure 1**).

### Forced Swimming Test

A modified FST procedure from the one described by Cryan et al. (2002) was used. On day 15 of chronic treatment (while estradiol or sertraline were still administered), a swim test session of 5 min was carried out with no pretest session. This approach is used in order to avoid the extended delay (2 weeks) between the first (pretest) and the second exposure (test) or the impact of drugs being present during the pretest session if pretest and test are separated by the usual 24 h. In addition, it has been shown that positive results with the drugs could be obtained even if no training session was carried out (Cryan et al., 2005; Benmansour et al., 2016). Rats were placed individually into a Plexiglas cylinder (21 × 46 cm) filled with 25°C water to a depth of 40-cm. As the older rats are heavier than the younger ones (4m-OVX+2w: 295 ± 2 g; 10m-OVX+2w: 314 ± 3 g; 14m-OVX+2w: 332 ± 6 g; 10m-OVX+4m: 362 ± 10 g; and 10m-OVX+8m: 400 ± 11 g) the depth of the water in the tank was adjusted so as not to compromise their swimming in the FST, e.g., 40 cm instead of 30 cm such that animals could not place their rear paws on the bottom without being totally submersed. Behaviors during the 5 min swim were recorded by a video camera positioned above the tank. A time sampling technique was employed whereby the predominant behavior in each 5 s bin of the test was analyzed. Climbing was defined as upward-directed movements of the forepaws along the side of the swim chamber. Swimming was defined as active movements (usually horizontal) throughout the swim chamber, which also include crossing into another quadrant. Immobility is assigned when no active movement other than that necessary to keep the rat’s head above the water. The rater was blind with respect to the experimental conditions being scored.

### Serum Levels of Sertraline and Its Metabolite

At the end of the FST session serum was collected from all treatment groups. Serum concentrations of sertraline and desmethyl-sertraline were determined by high performance liquid chromatography (HPLC) as described previously (Benmansour et al., 1999).

### Statistical Analysis

**FIGURE 2:**
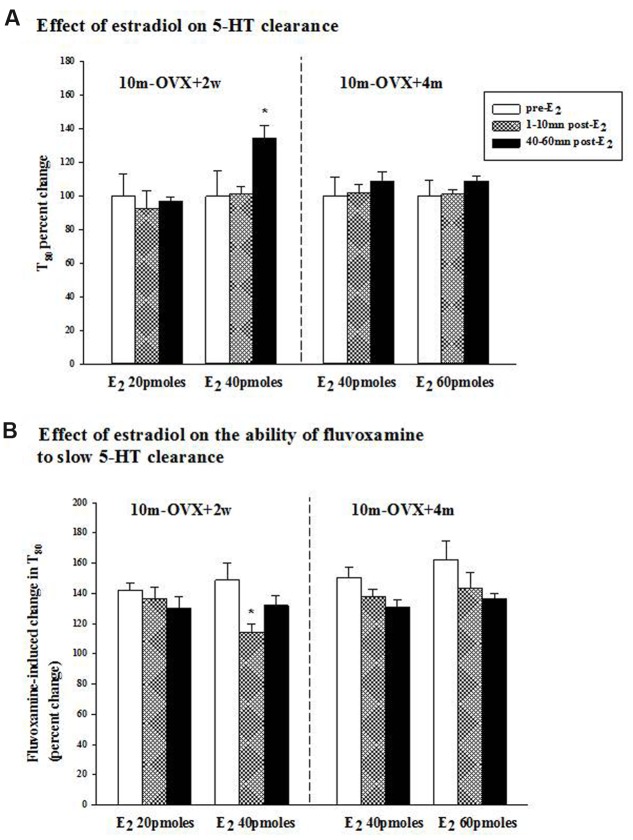
**Effects of acute estradiol administration on serotonin transporter (SERT) function in middle-aged OVX rats at different interval times post-OVX.** Estradiol (40 or 60 pmol) was locally applied into the CA3 region of the hippocampus of 10m-OVX+2w and 10m-OVX+4m and its effects measured at an early time point (1–10 min) and later time point (40–60 min) post-E_2_ administration on: **(A)** 5-HT clearance: the 5-HT clearance time parameter, T_80,_ was analyzed pre-E_2_, 1–10 min post-E_2_ and 40–60 min post-E_2_. Bar and brackets represent the T_80_ value as a percentage of the pre-treatment value ± SEM (*n* = 7–8). ^∗^*p* < 0.05, Kruskal–Wallis one-way ANOVA on ranks, followed by Dunn’s test comparing percent change in T_80_ values 1–10 min and 40–60 min post-E_2_ with the percent change in T_80_ value in the corresponding pre-E_2_ value. **(B)** The ability of fluvoxamine to slow 5-HT clearance: as shown by an increase in the T80 value. Fluvoxamine (4× the amount of 5-HT) was pressure ejected 60–90 s before the second application of 5-HT. Bar and brackets represent the percent change in the T_80_ value after fluvoxamine ± SEM (*n* = 6–10). ^∗^*p* < 0.05, Kruskal–Wallis one-way analysis of variance on ranks, followed by Dunn’s test comparing percent change in the T_80_ value post-fluvoxamine 1–10 min and 40–60 min after E_2_ with fluvoxamine’s percent change in the T_80_ value of the corresponding pre-E_2_ value.

**FIGURE 3:**
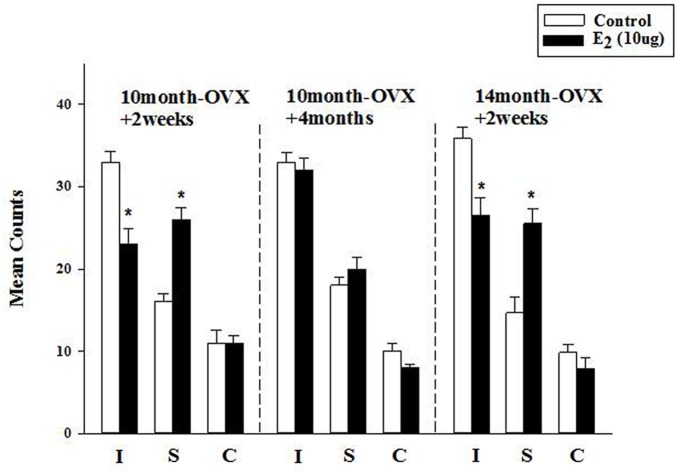
**Effect of long-term treatment with estradiol in the FST.** OVX rats (10m-OVX+2w, 10m-OVX+4m, and 14m-OVX+2w) were treated for 2 weeks with estradiol (10 μg) via subcutaneous osmotic minipumps, as described in the Section “Materials and Methods.” Mean counts for immobility, swimming and climbing behaviors were sampled every 5 s of the swim test period. Bars and brackets represent the mean value ± SEM, *n* = 6–8/group. Immobility (I): Two-way ANOVAs were carried out between: (1) age and treatment (10m-OVX+2w and 14m-OVX+2w groups) and 2) between time post-OVX and treatment (10m-OVX+2w, 10m-OVX+4m). When comparing age/treatment, two-way ANOVA showed a significant main effect for treatment [*F*_(1,24)_ = 28.23, *p* < 0.001] but no significant effect for age or for interaction between age × treatment. When comparing time post-OVX/treatment, two-way ANOVA showed a significant main effects for treatment [*F*_(1,26)_ = 11.47, *p* < 0.01], for time post-OVX [*F*_(1,26)_ = 8.13, *p* < 0.01] and for interaction between time post-OVX × treatment [*F*_(1,26)_ = 8.13, *p* < 0.01]. Dunnett’s *post hoc* analysis was carried out;^∗^*p* < 0.001, comparing treatment with E_2_ within 10m-OVX+2w and within 14m-OVX+2w with their respective controls. Swimming (S): Two-way ANOVAs were carried out between: (1) age and treatment (10m-OVX+2w and 18m-OVX+2w groups) and (2) between time post-OVX and treatment (10m-OVX+2w, 10m-OVX+4m). When comparing age/treatment, two-way ANOVA showed a significant main effect for treatment [*F*_(1,24)_ = 46.18, *p* < 0.001] but no significant effect for age or for interaction between age × treatment. When comparing time post-OVX/treatment, two-way ANOVA showed a significant main effects for treatment [*F*_(1,26)_ = 24.39, *p* < 0.001] and for interaction between time post-OVX × treatment [*F*_(1,26)_ = 8.68, *p* < 0.01] but no effect for time post-OVX. Dunnett’s *post hoc* analysis was carried out; ^∗^*p* < 0.001, comparing treatment with E_2_ within 10m-OVX+2w and within 14m-OVX+2w with their respective controls. Climbing (C): Two-way ANOVAs were carried out between age and treatment and between time post-OVX and treatment. There were no significant effects in any of the analyses.

**FIGURE 4:**
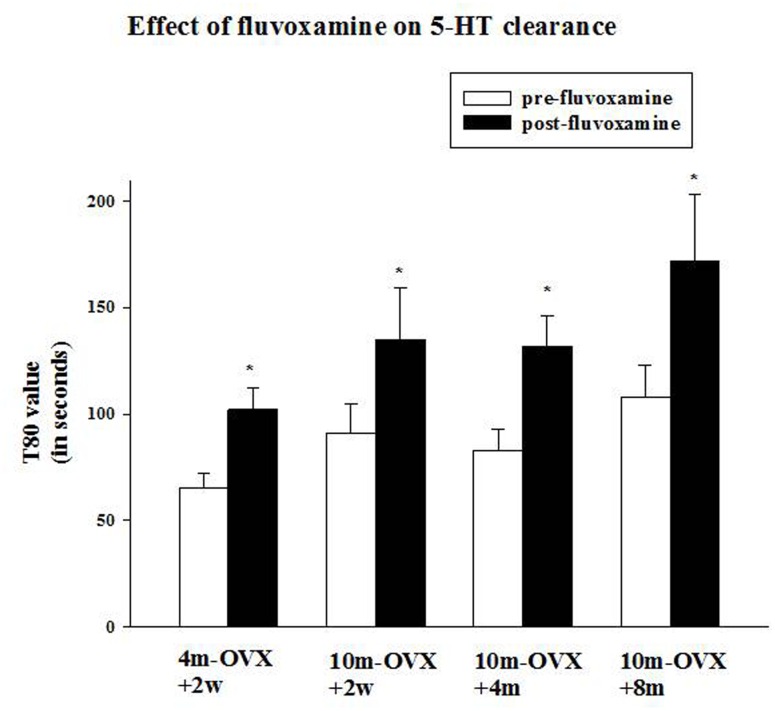
**Effects of acute fluvoxamine on SERT function of young adult OVX rat or middle-aged OVX rats at different interval times post-OVX.** Electrochemical recordings were carried out in the CA3 region of the hippocampus of 4m-OVX+2w, 10m-OVX+2w, 10m-OVX+4m, 10m-OVX+8m. Fluvoxamine (4× the amount of 5-HT) was pressure ejected 60–90 s before the second application of 5-HT. Bar and brackets represent mean T80 value (in seconds) before and after fluvoxamine application ± SEM (*n* = 6–9); ^∗^*p* < 0.01, paired Student’s *t*-test comparing post-fluvoxamine value with pre-fluvoxamine value for each group. One-way ANOVA comparing T80 values pre-fluvoxamine and post-fluvoxamine values in all groups reveals no differences among groups in these two values.

**FIGURE 5:**
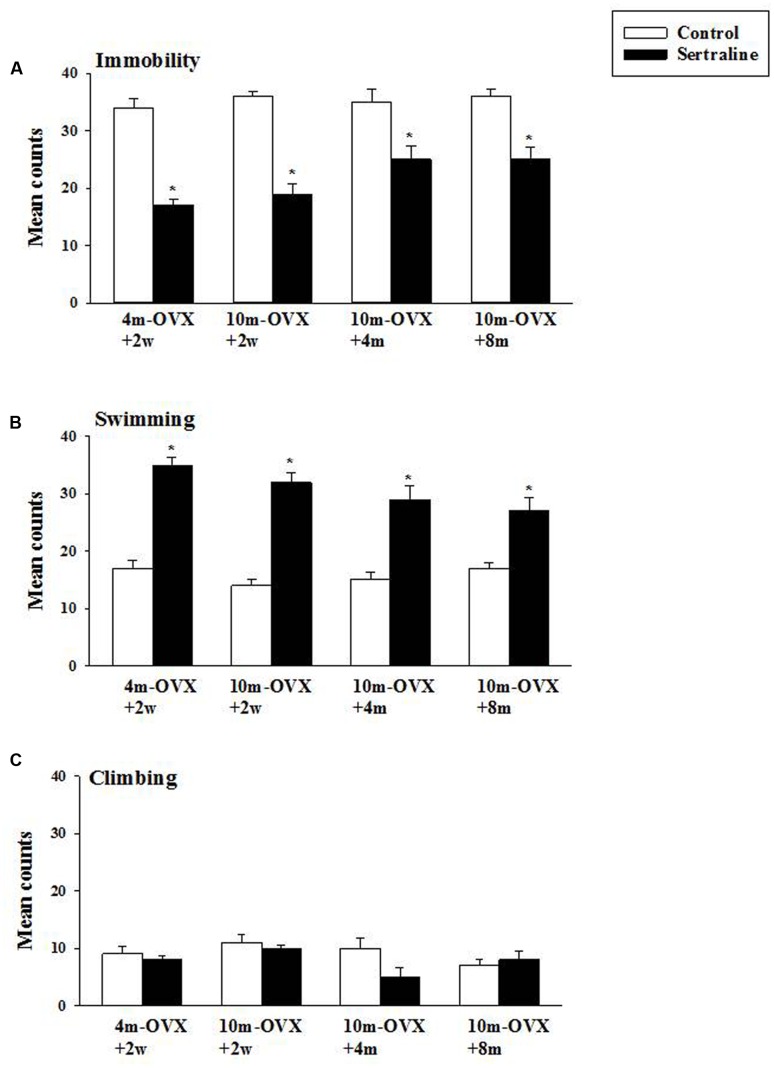
**Effect of chronic treatment with sertraline in the FST.** OVX rats (4m-OVX+2w, 10m-OVX+2w, 10m-OVX+4m, 10m-OVX+8m) were treated for 2 weeks with sertraline via subcutaneous osmotic minipumps, as described in the Section “Materials and Methods.” Mean counts for immobility, swimming and climbing behaviors were sampled every 5 s of the swim test period. Bars and brackets represent the mean value ± SEM, *n* = 8/group. **(A)** Immobility: Two-way ANOVAs were carried out between: (1) age and treatment (4m-OVX+2w and 10m-OVX+2w groups) and (2) between time post-OVX and treatment (10m-OVX+2w, 10m-OVX+4m, and 10m-OVX+8m groups). When comparing age/treatment, two-way ANOVA showed a significant main effect for treatment [*F*_(1,28)_ = 169.53, *p* < 0.001] but no significant effect for age or for interaction between age × treatment. When comparing time post-OVX/treatment, two-way ANOVA showed a significant main effect for treatment [*F*_(1,42)_ = 70.48, *p* < 0.001] but no significant effect for time post-OVX or for interaction between time post-OVX × treatment. Dunnett’s *post hoc* analysis was carried out; ^∗^*p* < 0.001, comparing 10m-OVX+2w with 4m-OVX+2w and 10m-OVX+4m and 10m-OVX+8m with 10m-OVX-2 weeks. **(B)** Swimming: Two-way ANOVAs were carried out between (1) age and treatment (4m-OVX+2w and 10m-OVX+2w groups) and (2) between time post-OVX and treatment (10m-OVX+2w, 10m-OVX+4m, and 10m-OVX+8m groups). When comparing age/treatment, two-way ANOVA showed a significant main effect for age [*F*_(1,28)_ = 5.24, *p* < 0.05] and for treatment [*F*_(1,28)_ = 178.80, *p* < 0.001] but no significant effect for interaction between age × treatment. When comparing time post-OVX/treatment, two-way ANOVA showed a significant main effect for treatment [*F*_(1,42)_ = 70.48, *p* < 0.001] but no significant effect for time post-OVX or for interaction between time post-OVX × treatment. Dunnett’s *post hoc* analysis was carried out;^∗^*p* < 0.001, comparing 10m-OVX+2w with 4m-OVX+2w and 10m-OVX+4m and 10m-OVX+8m with 10m-OVX-2 weeks. **(C)** Climbing: Two-way ANOVAs were carried out between age and treatment and between time post-OVX and treatment. There were no significant effects in any of the analyses.

Data were analyzed using SigmaStats (Systat Software Inc, San Jose, CA, USA). Kruskal–Wallis one-way analysis of variance (ANOVA) on ranks, followed by Dunn’s *post hoc* analysis was used to compare percent change in T80 values caused either by E_2_, fluvoxamine, or E_2_ plus fluvoxamine (**Figure 2**). Two-way ANOVA followed by Dunnett’s analysis was used to compare (1) effect of age or time post-OVX within the vehicle and within the E_2_ or the sertraline groups, and (2) effect of E_2_ or sertraline within the age groups and within time post-OVX groups (**Figures 3** and **5**; **Table 1**). Paired Student’s *t*-test was used when comparing T80 value post-fluvoxamine with T80 value pre-fluvoxamine in the same animal (**Figure 4**). One-way ANOVA was also used in **Figure 4** comparing T80 values pre-fluvoxamine and values post-fluvoxamine among all groups. Only when there was a significant main effect and/or interaction effect in the ANOVA’s were *post hoc* analyses carried out. Significance was determined at *p* < 0.05.

**Table 1:** Serum levels of sertraline.

	4m-OVX+2w	10m OVX+2w	10m-OVX+4m	10m-OVX+8m
Sertraline (ng/ml)	316^a^ ± 28	355 ± 46	609 ± 165	238 ± 29
Desmethyl-sertraline/sertraline (as a ratio)	0.416 ± 0.07	0.533 ± 0.04	0.644 ± 0.05	0.472 ± 0.06


*^a^Mean ± SEM.*

*Serum concentration of sertraline and desmethyl-sertraline were determined after 2 weeks treatment with sertraline (10 mg/kg/day) by subcutaneous osmotic minipumps implant. Also represented in the table is the ratio of desmethyl-sertraline/sertraline.*

*A two-way ANOVA analysis age/treatment and time post-OVX/treatment revealed no significant effect.*

## Results

### Effects of Local Application of Estradiol on SERT Function

The clearance time parameter, T80, derived from the generated 5-HT electrochemical signal, was analyzed in this study and is represented in **Figure 1**. In 4-month OVX rats, E_2_ (20 pmol) was shown previously to produce both early (1–10 min post-E_2_) and late (40–60 min post-E_2_) effects, i.e., it increased the T80 value (a measure of 5-HT clearance) and also blocked the fluvoxamine-induced increase in the T80 value (Benmansour et al., 2009, 2012). At that same dose in 10-month OVX rats, E_2_ had no effect on 5-HT clearance nor did it alter the ability of fluvoxamine to slow 5-HT clearance (**Figures 2A,B**, left panel). However, a higher dose of E_2_ (40 pmol) increased the T80 value but this effect was only seen 40–60 min post-application of E_2_ (**Figure 2A**, left panel). Fluvoxamine significantly increased the T80 value (to 140 ± 6% of the control value) in middle aged OVX rats. Administration of 20 pmol of E_2_ produced no inhibition of the fluvoxamine effect (**Figure 2B**, left panel). However, the higher amount of E_2_, 40 pmol, did reduce significantly the effect of fluvoxamine at 1–10 min after E_2_ application but not at 40–60 min post-E_2_ (**Figure 2B**, left panel). By contrast after 4 months of hormonal depletion, 40 pmol E_2_ had no effect on serotonin clearance nor did it alter the ability of fluvoxamine to slow 5-HT clearance in 10–month-old OVX rats. Increasing the dose to 60 pmol also did not produce any effects on the parameters measured (**Figures 2A,B**, right panel).

### Effect of Long-Term Treatment with Estradiol in the FST

The FST was used to investigate the longer-term effects of E_2_, administered subcutaneously via osmotic minipumps for 2 weeks. In 4-month OVX rats, it was shown previously that long-term E_2_ (5 μg) induced an AD-like effect in the FST (Benmansour et al., 2016). In 10-month OVX rats, E_2_ (5 μg) did not change immobility or swimming behavior in the FST as compared to that measured in control rats (data not shown). At a higher dose (10 μg), E_2_ induced a significant decrease in immobility that was associated with a significant increase in swimming behavior (**Figure 3**, first panel). However, E_2_ (10 μg) had no effect in 10-month OVX rats when the treatment was started 4 months post-OVX as opposed to 2 weeks post-OVX (**Figure 3**, second panel). By contrast, significant AD-like effects were obtained in 14-month OVX rats tested 2 weeks after OVX (**Figure 3**, third panel), thereby demonstrating that the lack of AD-like effects of E_2_ is due to the 4-month hormone withdrawal and not to an age effect.

### Effect of Local Application of an SSRI on SERT Function

This experiment was designed to examine the ability of the SSRI fluvoxamine to inhibit SERT function in 10-month-old middle-aged OVX rats in comparison to its effects in 4-month-old adult OVX rats and to determine in the middle-aged rats if the time after OVX when fluvoxamine is tested would influence its effects. As expected, fluvoxamine prolonged the clearance time of 5-HT in 4m-OVX+2w rats as shown by a significant increase in the clearance time T80 value post-fluvoxamine compared with the pre-fluvoxamine value (**Figure 4**). This is consistent with our previous data in young adult OVX rats (Benmansour et al., 2009, 2012, 2014). Similarly, fluvoxamine significantly increased the T80 value in 10m-OVX+2w, 10m-OVX+4m as well as 10m-OVX+8m when compared to their pre-fluvoxamine T80 values (**Figure 4**). Thus, fluvoxamine blocked SERT function in all the groups with the effect not dependent on the time elapsed after OVX.

### Effect of Chronic Treatment with Sertraline in the FST

The behavioral effects of chronic treatment with the SSRI sertraline in the FST were examined in young adult and middle-aged rats at short or longer times after OVX. As expected and shown previously, in 4m-OVX+2w rats, chronic treatment with sertraline induced an AD-like behavior in the FST, i.e., it significantly decreased immobility and increased swimming behaviors as compared to that in control rats. Similarly, chronic treatment with sertraline significantly decreased immobility and increased swimming behaviors in middle-aged rats at 2 weeks, 4 months, or 8 months post-OVX (**Figure 5**). None of the treatments altered climbing behavior. Although there was a trend for the effects of sertraline (on both immobility and swimming) to be of smaller magnitude in the 10m-OVX+4m and the 10m-OVX+8m than the other groups, this difference did not reach significance. There was no change in baseline behavior within groups (**Figure 5**).

In addition serum levels of sertraline and its metabolite desmethyl-sertraline were analyzed in each group after conclusion of the FST. Neither sertraline levels nor the ratio of desmethyl-sertraline/sertraline were significantly different in any of the experimental groups. Although there was a trend for both measurements to be higher in the 10m-OVX+4m than any of the other groups, this was not statistically significant (**Table 1**).

## Discussion

These data show that (1) a higher dose of estradiol was necessary to induce AD-like effects in middle-aged OVX rats as compared to those needed in young adult OVX rats; (2) treatment with SSRIs induced an AD-like effect in the middle-aged OVX rats that was similar to that seen in younger adult OVX rats; (3) AD-like effects of estradiol are dependent on the time interval post-ovariectomy whereas the effects of SSRIs are not. Moreover, such results are similar irrespective of whether they were obtained using chronoamperometry to measure SERT function directly in the CA3 region of the hippocampus or the FST to measure it indirectly.

Sensitivity to acute as well as long-term treatment with estradiol decreased with age in OVX rats (**Figures 2** and **3**). This has been demonstrated previously. Whereas acute treatment with estradiol both at 5 and 10 μg produced an AD-like effect in 3–month-old OVX rats, only 10 μg had an effect in 14-month-old OVX rats (Recamier-Carballo et al., 2012). In addition, differences exist in the acute effects of E_2_ in the younger and older rats. In young adult OVX rats, E_2_ had both rapid as well as effects that were manifest at a later time on both slowing 5-HT clearance and on blocking the ability of fluvoxamine to slow 5-HT clearance (Benmansour et al., 2009, 2012). The AD-like effect of E_2_ (i.e., blockade of 5-HT clearance) is due to ERβ activation whereas E_2_’s inhibition of fluvoxamine’s ability to slow serotonin clearance is mediated through the ERα receptor subtype (Benmansour et al., 2012). In middle-aged OVX rats, the effects of E_2_ on fluvoxamine-induced blockade of 5-HT clearance were rapid whereas its own effects on 5-HT clearance were manifest later in time (**Figure 2**).

Several mechanisms could explain these differential effects as well as the reduced sensitivity to estradiol with aging, e.g., subcellular redistribution of ERα and ERβ in middle age and/or an age-dependent decline in the number of available ERs. Studies using aged female animals have shown that the expression of both ERα and ERβ in the brain change with age in a region specific manner for each receptor subtype (Foster, 2012); however, both ER subunit proteins decreased in the hippocampus of aged female rats (Yamaguchi and Yuri, 2014). This could account, at least partially, for the reduced potency of estradiol observed in our experiments using chronoamperometry. In addition to the age-related changes in ER expression, the ability of estrogen to regulate ER expression changes with age and is different in each brain area for each ER subtype (Yamaguchi and Yuri, 2014). It is also possible that a decline in ERα function, rather than a loss of receptor expression, could mediate a decrease in estrogen’s effects with advanced age. Aging was shown to be associated with the expression of dominant-negative ERα splice variants that reduced the ability of estradiol treatments to preserve cognition (Bohacek and Daniel, 2009). Little is known how ER expression changes in the hippocampus of aging humans. However, and importantly, elderly women are more likely to express ERα splice variants that can act as dominant negative regulators, reducing or inhibiting ERα-mediated transcriptional activity (Ishunina and Swaab, 2009).

Similarly, several ERβ splice variants have been identified in the rat hippocampus. One of these, rERβ2, has a low binding affinity for estradiol and the ability to interact with coactivators such that it functions as a dominant negative receptor in the rat (Weiser et al., 2008). rERβ2 expression increased in the brains of intact rats with age and estropause (Wang et al., 2012). Finally, estrogen signaling possibly involving either cAMP response element-binding protein (CREB) or extracellular signal-regulated kinases (ERK), also changes with age. Age-related decreases in rapid E_2_ responses are observed for E_2_-mediated growth in synaptic transmission and facilitation of long-term potentiation (Foy et al., 2008), and activation of ERK (Bi et al., 2003). Also, the levels of total or phosphorylated CREB are reduced in the hippocampus of old rats (Kudo et al., 2005). The effect of aging on CREB seems to be specific for the hippocampus because changes in pCREB were not observed in the frontal cortex of old rats (Xu et al., 2010). All these factors, then, may be in play in accounting for our observations of diminished effects of estradiol in 10-month OVX rats as compared to younger OVX rats.

Studies on the effect of AD treatment using older female animals are scarce, with most having tested only the effects of acute treatment with ADs. Consistent with our data, acute fluoxetine (10 mg/kg) treatment produced an analogous response in young adult and middle-aged OVX rats in the FST, although a lower dose 2.5 mg/kg reduced immobility in younger rats but not in middle-aged females (Recamier-Carballo et al., 2012; Fernandez-Guasti et al., 2016). In contrast, citalopram (up to 10 mg/kg) given sub-acutely to middle-aged OVX rats had no effect in the FST (Vega Rivera et al., 2016), although it did produce an AD-like effect in middle-aged OVX rats that had been subjected to chronic mild stress (Romano-Torres and Fernandez-Guasti, 2010). Chronic treatment with fluoxetine had no effect in middle-aged female mice (Li et al., 2015).

Modulation of 5-HT neurotransmission has long been a primary pharmacological target for the treatment of depression and more recently anxiety disorders by SSRIs. SSRIs enhance synaptic 5-HT action by blocking the reuptake of 5-HT (Schloss and Williams, 1998). Global enhancement of serotonin neurotransmission may activate all subtypes of serotonin receptors in brain, whereas each 5-HT receptor subtype has different and specific functions in defined brain regions (Polter and Li, 2011). The process of aging has complex effects on 5-HT neurotransmission throughout central and peripheral systems. Although there are no clear alterations in the number of 5-HT neurons, accumulated evidence suggests aging compromises 5-HT neurotransmission as well as producing altered expression of SERT and 5-HT receptors in multiple brain regions (Rodriguez et al., 2012; Fidalgo et al., 2013). Contradictory results have been reported on age-related changes in SERT, with decreases, increases or no change being described (Brunello et al., 1985; Severson et al., 1985; Owen et al., 1986; Marcusson et al., 1987; Arranz et al., 1993; Yamamoto et al., 2002). Multiple studies have demonstrated a decrease in serotonin receptor 1A (5-HT1A), serotonin receptor 2A (5-HT2A) receptor binding sites/affinity, protein expression and/or gene expression in the brain with increasing age (Meltzer et al., 1998, 2001; Tauscher et al., 2001); however, this has not always been replicated (Parsey et al., 2002). This variability could be due to the species, the brain area or the measurement techniques used to quantify SERT and/or 5-HT receptors (Rodriguez et al., 2012). Little is known, however, about age-related changes in serotonergic function in females. In this study, the effect of local application of an SSRI into the CA3 region of the hippocampus on SERT function was similar in young adult and middle-aged OVX rats (**Figure 4**). Also similar was the effect of sertraline in the FST in all the groups (**Figure 5**). Thus, two different measures of the effect of SSRIs on SERT function gave the same result, namely that effects were similar in younger and middle aged OVX rats. Pharmacokinetic changes produced by age could modify the drug effects. The most significant change is an age-dependent reduction of renal excretion, caused by deficient glomerular filtration (Turnheim, 2003; Mangoni and Jackson, 2004). As a consequence, drug serum levels tend to increase, producing a stronger pharmacological effect. This was not the case in our study as in middle-aged OVX rats levels of sertraline and its metabolite were not significantly different from those measured in younger OVX rats (**Table 1**).

Clinical data have been equivocal whether estrogen therapy in postmenopausal women benefits cognition (Maki, 2012; Daniel, 2013). A reevaluation of the role of estrogens as neuroprotective agents led to the development of “the critical period” or “window of opportunity” hypothesis of estrogen effects (Rocca et al., 2011). This proposes that estrogen therapy is only beneficial if administration is begun during a time window in close proximity to menopause (Resnick and Henderson, 2002). An initial study in rats provided the first suggestion of a critical period for the efficacy of estrogen (Gibbs, 2000). Initiation of estradiol treatment immediately or 3 months following ovariectomy in middle-aged rats enhanced performance on a delayed matching-to-position maze task whereas no enhancement of performance was evident if estradiol administration was begun 10 months after ovariectomy (Gibbs, 2000). Other data also indicated that beneficial effects of estradiol on cognition, cholinergic function, and hippocampal plasticity were obtained only when hormone treatment was initiated at relatively early time points after ovariectomy (Daniel, 2013).

Many studies have examined the critical period hypothesis with respect to cognition but only a few clinical studies have investigated the effect of the time of initiation of ERT and/or AD therapy after menopause in mood disorders (see Craig, 2013). It was reported that ERT improved mood in pre-menopausal but not post-menopausal women (Cohen et al., 2003). There have been very few preclinical studies as well. A recent study showed that an AD-like effect induced by acute treatment with ethinyl-estradiol treatment was found after 1 week but not 3 weeks post-OVX (Fernandez-Guasti et al., 2016). In middle-aged OVX rats, estradiol had an AD-like effect in the FST when given at a short time post-OVX but not when given 5–6 months later (Walf et al., 2009; Kiss et al., 2012; Wang et al., 2012). Consistent with this, our data show that the AD-like effect of estradiol in middle aged rats was present only when treatment was started 2 weeks post-OVX but not 4 months post-OVX (**Figures 2** and **3**). In addition, this effect was confirmed to be due to the longer time interval post-OVX and not to aging as 14-month OVX rats displayed an AD-like effect when tested 2 weeks post-OVX in the FST (**Figure 3**). Emerging data implicate a decline in ERα expression or function in brain resulting from long-term hormone deprivation as a basis for the existence of the critical period (Foster, 2012). In addition it was suggested that the C terminus of Hsc70-interacting protein-mediated degradation of hippocampal ERα may serve as a molecular mechanism for the critical therapeutic window for post-menopausal estrogen therapy (Zhang et al., 2011). Another potential mechanism may be due to increased expression of the dominant negative ERβ isoform, ERβ2, which correlated with a significant decrease in swim time in the FST and appears to be a crucial determinant responsible, at least in part, for the loss of estrogen response after protracted absence of ovarian hormones in a rat model of menopause (Wang et al., 2012). Experiments examining such parameters will be necessary to determine the mechanisms responsible for the results obtained here after either acute or chronic treatment with estradiol.

This study also examined if prolonged estrogen deprivation may reduce the likelihood of SSRIs having an AD-like effect. It was found that SSRIs have the same efficacy at blocking SERT function and inducing AD-like effects in the middle-aged OVX rats tested 2 weeks, 4 months, or 8 months post-OVX (**Figures 4** and **5**). Only a few studies have investigated the critical window for AD effects in middle-aged rats as opposed to younger OVX rats. In one study, acute citalopram treatment had no effect in the FST in middle aged-rats independent of the post OVX interval. The authors concluded that age and endocrine condition are factors that contribute to decreased sensitivity to citalopram (Vega Rivera et al., 2016). Treatment with fluoxetine was reported to be efficacious in depressed middle-aged women (Amsterdam et al., 1999) and that pre- and post-menopausal women suffering from depression both responded to citalopram treatment (Kornstein et al., 2013). However, a poorer response to AD treatment in post-menopausal women compared to pre-menopausal women has also been reported (Pae et al., 2009).

## Conclusion

This study showed that the sensitivity to estradiol decreased in middle–aged OVX rats compared to younger adult OVX rats, whereas the AD-like effects of SSRIs was not diminished in middle-aged OVX rats compared to young adult OVX rats. The AD-like effect of estradiol, in contrast to that of SSRIs, is subject to a critical period. In addition despite the potential benefits of estradiol treatment, hormone replacement therapy presents unwanted health risks, particularly in older post-menopausal women (Lukes, 2008). Findings from the Women’s Health Initiative trial show that HRT in healthy postmenopausal women is associated with an elevated risk of breast cancer and coronary heart disease (Rossouw et al., 2002). In addition, the risk of breast cancer increases with longer exposure to HRT, limiting long-term use (Rossouw et al., 2002; Anderson et al., 2006). An additional disadvantage of estradiol treatment is that estradiol act as a non-selective ER agonist. ERα and ERβ appear to have opposing functions in several brain areas. As shown previously, ERβ mediates the AD-like effect of estradiol whereas ERα mediates its blockade of the AD-like effect of SSRIs (Benmansour et al., 2012); and that could result in diminishing therapeutic potential of estradiol. Thus, if our results in rats can be translated to the clinical situation, they indicate that SSRIs have essentially identical effects on the SERT in females regardless of age and the time of treatment initiation after menopause. However, if estradiol is to be tried in postmenopausal women suffering from depression, our results suggest that it might only be helpful to those in perimenopause or shortly after menopause begins.

## Author Contributions

SB contributed to the design, acquisition and interpretation of the data, and the draft of the manuscript. LA contributed to the acquisition and interpretation of the data and helped draft the manuscript. AF contributed to data analysis, interpretation, and provided intellectual contribution during drafting of the manuscript.

## Conflict of Interest Statement

Dr. Benmansour and Mr. Arroyo have no biomedical financial interests or potential conflicts of interest. Dr. Frazer has been on advisory boards for Cyberonics, Inc., H. Lundbeck A/S, and Takeda Pharmaceuticals America, Inc. and he has consulted and/or received research support for preclinical studies from Forest Research Institute, Eli Lilly and Company, Wyeth Pharmaceuticals, and H. Lundbeck A/S. No support for this study was received from any pharmaceutical company.
